# Residential Pesticides and Childhood Leukemia: A Systematic Review and Meta-Analysis

**DOI:** 10.1289/ehp.0900966

**Published:** 2009-07-29

**Authors:** Michelle C. Turner, Donald T. Wigle, Daniel Krewski

**Affiliations:** 1 McLaughlin Centre for Population Health Risk Assessment, Institute of Population Health; 2 Faculty of Graduate and Postgraduate Studies and; 3 Department of Epidemiology and Community Medicine, Faculty of Medicine, University of Ottawa, Ottawa, Canada;; 4 Risk Sciences International, Ottawa, Canada

**Keywords:** child, environmental exposure, leukemia, meta-analysis, pesticides

## Abstract

**Objective:**

We conducted a systematic review and meta-analysis of previous observational epidemiologic studies examining the relationship between residential pesticide exposures during critical exposure time windows (preconception, pregnancy, and childhood) and childhood leukemia.

**Data sources:**

Searches of MEDLINE and other electronic databases were performed (1950–2009). Reports were included if they were original epidemiologic studies of childhood leukemia, followed a case–control or cohort design, and assessed at least one index of residential/household pesticide exposure/use. No language criteria were applied.

**Data extraction:**

Study selection, data abstraction, and quality assessment were performed by two independent reviewers. Random effects models were used to obtain summary odds ratios (ORs) and 95% confidence intervals (CIs).

**Data synthesis:**

Of the 17 identified studies, 15 were included in the meta-analysis. Exposures during pregnancy to unspecified residential pesticides (summary OR = 1.54; 95% CI, 1.13–2.11; *I*^2^ = 66%), insecticides (OR = 2.05; 95% CI, 1.80–2.32; *I*^2^ = 0%), and herbicides (OR = 1.61; 95% CI, 1.20–2.16; *I*^2^ = 0%) were positively associated with childhood leukemia. Exposures during childhood to unspecified residential pesticides (OR = 1.38; 95% CI, 1.12–1.70; *I*^2^ = 4%) and insecticides (OR = 1.61; 95% CI, 1.33–1.95; *I*^2^ = 0%) were also positively associated with childhood leukemia, but there was no association with herbicides.

**Conclusions:**

Positive associations were observed between childhood leukemia and residential pesticide exposures. Further work is needed to confirm previous findings based on self-report, to examine potential exposure–response relationships, and to assess specific pesticides and toxicologically related subgroups of pesticides in more detail.

Leukemia is the most common form of childhood cancer in Canada and the United States, accounting for > 30% of new cancer cases (American Cancer Society 2009; Canadian Cancer Society/National Cancer Institute of Canada 2009). During 2000–2004, there were nearly 1,400 new cases of leukemia among children 0–14 years of age in Canada, with incidence rates highest among those 0–4 years of age (Agha et al. 2006; Canadian Cancer Society/National Cancer Institute of Canada 2009). Acute lymphoblastic leukemia (ALL) accounts for most (~ 80%) childhood leukemia cases, followed by acute myelogenous leukemia (AML) (Canadian Cancer Society/National Cancer Institute of Canada 2009). Although much progress in treating childhood leukemia has been achieved, treatment entails substantial morbidity, and elevated morbidity and mortality outcomes continue to be observed among survivors compared with children who have not developed the disease (MacArthur et al. 2007; Speechley et al. 2006).

Acute leukemias are heterogeneous, characterized by different genetic and chromosomal abnormalities, with differing frequency by age (Greaves 2002). The two-step model for childhood leukemia proposes that leukemia development occurs after both a first mutation, usually a chromosomal translocation occurring *in utero*, and a second mutation occurring after birth (Greaves 2002; Rossig and Juergens 2008). Children with Down syndrome experience an elevated risk for the disease (Alderton et al. 2006; Ross et al. 2005). Although a variety of environmental and chemical exposures have been suggested to play a role in the etiology of the disease, ionizing radiation remains the sole environmental risk factor established to date (Belson et al. 2007). Other potential risk factors that have received some attention in the scientific literature include parental smoking and alcohol consumption, electromagnetic field exposure, hydrocarbons, socioeconomic factors, immunity and infection, and pesticides (Belson et al. 2007; Greaves 2006; Infante-Rivard and El-Zein 2007; Lee et al. 2009; Rossig and Juergens 2008; Schuz and Ahlbom 2008).

Several studies examining the potential association between childhood leukemia and both parental occupational and residential pesticide exposure have been conducted over the past several decades, with positive associations observed (Infante-Rivard and Weichenthal 2007). Partly because of concerns surrounding potential adverse child health impacts, several Canadian provinces and municipalities have recently banned the cosmetic use of pesticides on public or private property (Arya 2005; Ontario Ministry of the Environment 2008). Similar bans are also being considered elsewhere.

Residential pesticide use is associated with elevated child exposures. Use of pyrethroid insecticides in the household was found to be a significant predictor of urinary pyrethroid metabolite levels in children in a recent longitudinal study (Lu et al. 2009). Child urinary concentrations of two organophosphorus pesticide metabolites (dimethyl and diethyl dialkylphosphate compounds) were found to be higher with parental garden pesticide use but not with pet treatment or indoor pesticide use in a Seattle study (Lu et al. 2001).

We conducted a systematic review and meta-analysis of previous observational epidemiologic studies examining the relationship between residential pesticide exposures during critical exposure time windows (preconception, pregnancy, and childhood) and childhood leukemia and explored potential methodological and clinical sources of heterogeneity in results. Although there have been previous reviews, none have included a quantitative synthesis of the results available to date. Results of an analysis examining the association between childhood leukemia and parental occupational pesticide exposure are presented in a separate, companion review (Wigle et al. 2009).

## Materials and Methods

This systematic review and meta-analysis was conducted according to a protocol designed by M.C.T. and D.T.W.

### Literature search

The search strategy was designed to identify previous observational epidemiologic studies examining the relationship between residential pesticide exposures during critical exposure time windows (preconception, pregnancy, childhood) and childhood leukemia. Preliminary searches using Ovid MEDLINE were conducted to inform the design of the final search strategy detailed below. An information specialist at the University of Ottawa was also consulted in finalizing the search strategy.

The search strategy was first developed to search the Ovid MEDLINE (1950–March week 3, 2009) and Ovid MEDLINE database of in process and other nonindexed citations (1950 to 31 March 2009) and then adapted to search the Ovid EMBASE (Excerpta Medica Database; 1980 to week 13 2009) (Ovid 2009), TOXNET (Toxicology Data Network; U.S. National Library of Medicine 2009) (through 31 March 2009), OpenSigle (2009) (through 31 March 2009), and ProQuest Digital Dissertations and Theses (2009) (through 31 March 2009). The following medical subject headings (MeSH) and key words were used:

- Exposure: exp Environmental Exposure/, exp Environmental Pollutants/, exp Pest Control/, exp Pesticides/, pesticid$.tw, herbicid$.tw, insecticid$.tw, fungicid$.tw
- Population: exp Child/, exp Adolescent/, exp Infant/, child$.tw, adolescen$.tw, infant?.tw, newborn?.tw, youth.tw, teenage$.tw
- Outcome: exp Hematologic Neoplasms/, exp Leukemia/, leuk?emia$.tw

Search terms were grouped according to the Boolean operators OR and AND. A complete depiction of the Ovid MEDLINE search strategy is given in Supplemental Material, Table 1 (available online at doi:10.1289/ehp.0900966.S1 via http://dx.doi.org).

All titles and abstracts identified were independently examined by two of us (M.C.T. and D.T.W.) in order to determine their potential suitability for inclusion in the systematic review. After this primary screen, the complete articles were obtained and the inclusion/exclusion criteria applied. Discrepancies were resolved by consensus. No language criteria were applied. Where abstracts were identified or further details required, particularly relating to the designation of pesticide exposure as residential or occupational, the corresponding author was contacted to ascertain further details of the study. In addition to searching the databases listed above, the reference lists of all included studies and journal Web sites were also hand searched; studies identified manually were evaluated in the same manner as above.

### Inclusion and exclusion criteria

Original epidemiologic studies of childhood leukemia using a case–control or cohort design with an assessment of at least one index of residential/household pesticide exposure/use were included here. Reports were excluded if they were review articles, ecologic studies, case reports, cluster investigations, or studies of adults or if they examined residential exposure or proximity to agricultural pesticides. Where there were multiple publications, the most relevant report was retained (usually the most recent).

### Data abstraction

After identification of all relevant studies, data abstraction was performed independently by the same two reviewers (M.C.T. and D.T.W.). A standard data abstraction form was prepared and piloted to collect relevant data related to referencing, study design, subject selection, exposure assessment, statistical analysis, and results. A single exposure index was identified for each original study and, where data were available, each combination of exposure time window and pesticide type (unspecified, insecticides, herbicides).

### Quality assessment

All included studies underwent independent quality assessment by the same two reviewers (M.C.T. and D.T.W.). We used a modified version of the Downs and Black (1998) checklist for the assessment of the methodological quality of randomized and nonrandomized studies of health care interventions [Supplemental Material, Table 2 (doi:10.1289/ehp.0900966.S1)]. Before conducting the quality assessment, the two reviewers discussed the individual items on the checklist to clarify their interpretation. No attempt was made to blind the reviewers of the authorship or publication status of the original studies. Differences in quality assessment were resolved by consensus.

### Analysis

We conducted meta-analyses using Review Manager (RevMan) version 5.0 (Nordic Cochrane Centre, Cochrane Collaboration, Copenhagen, Denmark). Generic inverse variance data were combined using random effects models to obtain a summary odds ratio (OR) and 95% confidence interval (CI) for the relationship between residential pesticide exposures (unspecified, insecticides, herbicides) and childhood leukemia by exposure time window (preconception, pregnancy, childhood). Heterogeneity across individual studies was quantified by the *I*^2^ statistic (Higgins et al. 2003). Low, moderate, or high degrees of heterogeneity may be approximated by *I*^2^ values of 25%, 50%, and 75%, respectively (Higgins et al. 2003). We conducted subgroup analyses according to total quality score (≥ median) and individual quality score components (> median for external validity and exposure measurement), study design (hospital-based or population-based case–control study), cell type (ALL, AML), location (indoor, outdoor), maternal residential pesticide use (vs. household use or exposure) only, year of publication (studies published in 2000 or later only), and publication status (studies published in the peer-reviewed literature only). Where multiple exposure indices were reported per exposure time window, pesticide type, and study, sensitivity analyses were undertaken using exposure or time window definitions different from those used in the main analysis. Finally, we also examined the impact of removing studies with extreme ORs or the highest weight in analysis, as well as removing individual studies in a sequential manner. Because of the small number of included studies, we assessed publication bias by visual inspection of inverted funnel plots, based on the main finding from all studies (Ioannidis and Trikalinos 2007).

## Results

### Study identification

**Figure 1:**
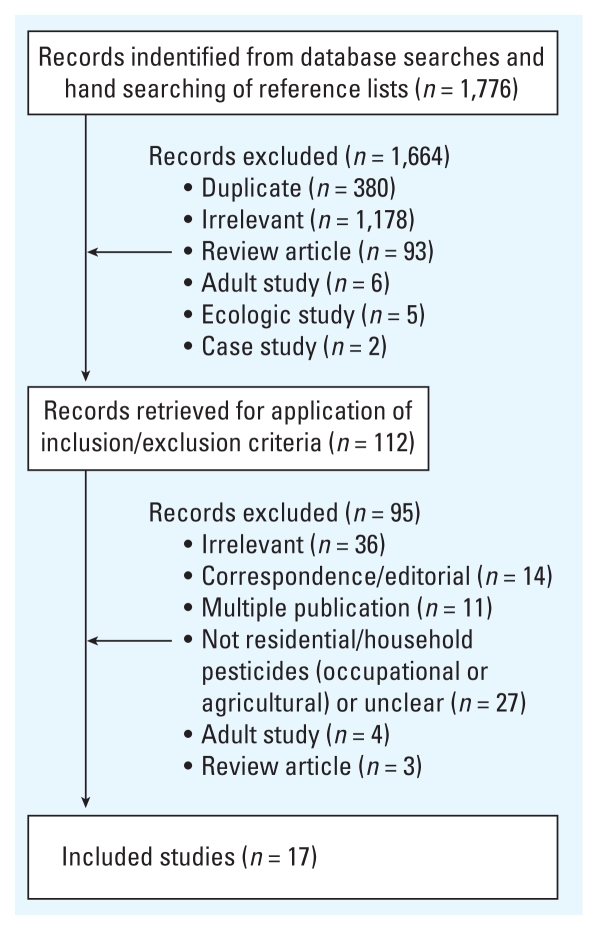
Study selection.

The results of the search strategy and study selection process are detailed in Figure 1. Of the 1,776 studies identified using our search algorithm, 112 were retained from the primary screening process. Most studies were excluded during primary screening because they were irrelevant (*n* = 1,178), a duplicate record (*n* = 380), or a review article (*n* = 93). After the secondary screening process, 17 studies were retained (listed in Table 1). Major reasons for exclusion during secondary screening were irrelevance (*n* = 36), examination of occupational or residential exposure to agricultural pesticides exposure only (or unclear) (*n* = 27), or a letter or editorial with no results presented (*n* = 14).

### Study characteristics

Of the 17 identified case–control studies, 6 were hospital based, 10 were population based, and 1 reported results separately for both hospital and population controls (Table 1). Studies were conducted in the United States, Canada, Mexico, Japan, France, Brazil, and Germany. Most of the studies were published in the peer-reviewed literature; however, three doctoral dissertations presented results not published elsewhere (Davis 1991; Dell 2004; Steinbuch 1994). Although most studies examined both ALL and acute nonlymphoblastic leukemia cases among children and adolescents up to a maximum age of 19 years, one study examined infantile acute leukemia (Pombo-de-Oliveira et al. 2006) and another examined both ALL and AML in children with Down syndrome (Alderton et al. 2006).

Studies varied in size, ranging from a total of 49 leukemia cases (with 7–25 cases ever exposed, depending on exposure category and time window) in the dissertation by Dell (2004) up to 1,184 cases (with 25–164 exposed) in the German study by Meinert et al. (2000). All studies conducted to date relied on parental reports of residential pesticide exposure or use inside or outside of the home, either by themselves or by professional exterminators [Supplemental Material, Appendix 1 (doi:10.1289/ehp.0900966.S1)]. Although most studies assessed use of, or exposure to, pesticides or specific pesticide subgroups (insecticides, herbicides, fungicides), some studies also attempted to collect information on pesticide names and formulations or on target organism (Davis 1991; Dell 2004; Infante-Rivard et al. 1999; Ma et al. 2002; Steinbuch 1994). Nine studies considered both residential and occupational pesticide exposures (Buckley et al. 1989; Dell 2004; Kishi et al. 1993; Lowengart et al. 1987; Meinert et al. 1996, 2000; Menegaux et al. 2006; Rudant et al. 2007; Steinbuch 1994), and the remaining eight studies were exclusively residential. Five studies clearly specified (or explicitly assumed) whether residential pesticide exposure during pregnancy was attributable to maternal use (Davis 1991; Lowengart et al. 1987; Ma et al. 2002; Menegaux et al. 2006; Rudant et al. 2007) as opposed to household use or exposure.

Virtually all studies assessed pesticide exposures during separate preconception, pregnancy, and childhood time windows; however, time window definitions differed somewhat by study (Appendix 1). Leiss and Savitz (1995) considered only the last 3 months of pregnancy. Ma et al. (2002) considered the first 3 years of age in a separate manner. Davis (1991) considered the first 6 months of age separately from the remainder of the childhood period. Some studies also combined results from different exposure time windows in analysis and reporting (Meinert et al. 1996, 2000; Rudant et al. 2007; Steinbuch 1994).

### Quality assessment

Quality scores are presented in Supplemental Material, Table 3 (doi:10.1289/ehp.0900966.S1). For hospital-based studies, total scores ranged from 7 to 12, with a median value of 9, of a possible maximum score of 20. For population-based studies, quality scores were higher, with a range of 9–14 and a median of 11. More recent studies tended to have higher quality scores. In assessing external validity, questions remained regarding the representativeness of subjects (both selected and participating), particularly for earlier hospital-based studies. Only Buckley et al. (1989) reported that interviewers were blind to case/control status; however, the ethics of such practices have also been questioned (Infante-Rivard et al. 1999). Because of the self-reported nature of exposure data, no study received a point for avoidance of bias from misclassification, since the possibility for differential misclassification remained. Only Dell (2004) and Ma et al. (2002) reported results for a clearly defined preconception exposure time window. There were few data regarding frequency or duration of pesticide use, with most studies reporting only “ever/never” use of/exposure to the pesticide of interest. Six studies attempted to examine potential exposure–response relationships (Alderton et al. 2006; Buckley et al. 1989; Dell 2004; Infante-Rivard et al. 1999; Ma et al. 2002; Meinert et al. 2000). Although confounding is difficult to assess because there are few established risk factors for childhood leukemia, most studies examined or adjusted for at least a range of sociodemographic and maternal characteristics. Four studies, however, explicitly assessed the potential confounding influence of maternal or childhood X-ray exposure (Dell 2004; Fajardo-Gutierrez et al. 1993; Kishi et al. 1993; Lowengart et al. 1987).

### Publication bias

To assess the possibility of publication bias, we examined the main findings from all included studies in an inverse funnel plot [Supplemental Material, Figure 1 (doi:10.1289/ehp.0900966.S1)]. Although limited by the small number of individual studies, there was some evidence for asymmetry, with a lack of small studies found with effect sizes smaller than those from larger studies. Asymmetry may also be due to a range of other factors, including study quality, methodological differences, or the study populations per se. We attempted to identify all relevant original studies possible, including three doctoral dissertations (Davis 1991; Dell 2004; Steinbuch 1994) and two studies published in a language other than English (Fajardo-Gutierrez et al. 1993; Kishi et al. 1993).

### Data synthesis

Of the 17 identified studies, we excluded two from the quantitative data synthesis due to a lack of CIs (Schwartzbaum et al. 1991) or a unique study population (Down syndrome cases only) (Alderton et al. 2006). Supplemental Material, Appendix 2 (doi:10.1289/ehp.0900966.S1) lists the individual studies included in each overall and subgroup analysis by exposure time window and pesticide type.

Preconceptional household use of unspecified residential indoor (summary OR = 1.53; 95% CI, 0.98–2.39; *I*^2^ = 0%) and outdoor (OR = 1.69; 95% CI, 1.03–2.77; *I*^2^ = 0%) pesticides was positively associated with childhood leukemia based on the two available studies (Dell 2004; Ma et al. 2002). We also found a significant positive association with preconceptional residential insecticide use (OR = 1.92; 95% CI, 1.34–2.74; *I*^2^ = 0%) in the same two studies.

**Figure 2:**
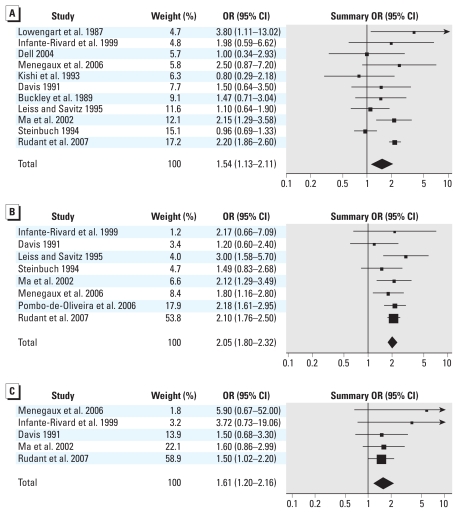
Analysis of the association between childhood leukemia and exposure to (*A*) unspecified residential pesticides during pregnancy, (*B*) residential insecticides during pregnancy, and (*C*) residential herbicides during pregnancy. Squares indicating ORs from individual studies are proportional in size to the weight assigned to each estimate.

Exposure to unspecified residential pesticides during pregnancy had a significant and positive association with childhood leukemia when combining results from 11 studies (OR = 1.54; 95% CI, 1.13–2.11; *I*^2^ = 66%), although the combined estimate had substantial heterogeneity (Figure 2A, Table 2). The magnitude of the positive association increased somewhat and heterogeneity was reduced when we examined ALL only (OR = 2.04; 95% CI, 1.54–2.68; *I*^2^ = 19%), indoor use of unspecified pesticides (OR = 1.86; 95% CI, 1.25–2.77; *I*^2^ = 9%), maternal use of unspecified pesticides (OR = 2.07; 95% CI, 1.62–2.64; *I*^2^ = 19%), and studies published in the peer-reviewed literature only (OR = 1.81; 95% CI, 1.37–2.39; *I*^2^ = 36%). We observed the largest OR for studies published in or since the year 2000 (OR = 2.17; 95% CI, 1.85–2.53; *I*^2^ = 0%).

Exposure to residential insecticides during pregnancy was associated with a significant increase in risk of childhood leukemia, when combining the results from eight studies (OR = 2.05; 95% CI, 1.80–2.32; *I*^2^ = 0%), with little evidence of heterogeneity (Figure 2B, Table 3). The summary OR changed little according to total study quality, individual quality components, hospital- versus population-based design, or publication status. The association was somewhat stronger for studies of ALL (OR = 2.14; 95% CI, 1.83–2.50; *I*^2^ = 0%) compared with AML (OR = 1.85; 95% CI, 1.29–2.64; *I*^2^ = 0%) and for indoor use (OR = 1.90; 95% CI, 0.61–2.23; *I*^2^ = 0%) compared with outdoor use (OR = 1.54; 95% CI, 0.86–2.74; *I*^2^ = 36%), although they are based on fewer studies.

Exposure to residential herbicides during pregnancy also had a significant positive association with childhood leukemia (OR = 1.61; 95% CI, 1.20–2.16; *I*^2^ = 0%) when combining the results from five studies (Figure 2C, Table 4). Again, we observed little difference in the summary OR according to study quality, study design, or publication status. The combined relative risk estimate increased somewhat for ALL (OR = 1.73; 95% CI, 1.28–2.35; *I*^2^ = 0).

Results for the pregnancy exposure time window were fairly robust to sensitivity analyses: removing studies with extreme ORs or with the highest weight or including additional studies with wide or ill-defined exposure time windows. However, removing the study of AML by Steinbuch (1994) from the unspecified pesticide analysis did result in a somewhat stronger association (OR = 1.74; 95% CI, 1.36–2.24; *I*^2^ = 31%).

**Figure 3:**
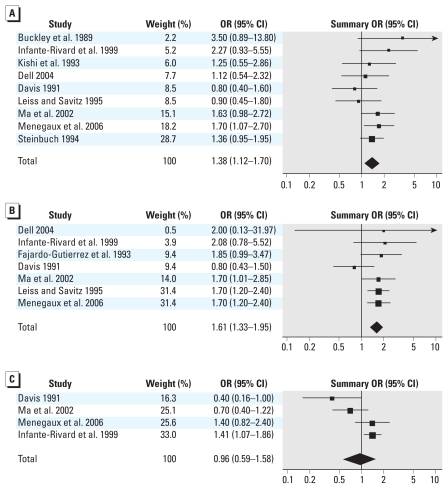
Analysis of the association between childhood leukemia and exposure to (*A*) unspecified residential pesticides during childhood, (*B*) residential insecticides during childhood, and (*C*) residential herbicides during childhood. Squares indicating ORs from individual studies are proportional in size to the weight assigned to each estimate.

Results for residential pesticide exposure during childhood were somewhat similar but were attenuated compared with those for pregnancy (Figure 3A, Table 2). We found a significant positive association between residential unspecified pesticide exposure during childhood and childhood leukemia (OR = 1.38; 95% CI, 1.12–1.70; *I*^2^ = 4%), with little heterogeneity. The magnitude of the association was somewhat stronger for indoor use (OR = 1.56; 95% CI, 1.02–2.39; *I*^2^ = 7%), studies published in or since the year 2000 (OR = 1.55; 95% CI, 1.14–2.12; *I*^2^ = 0%), and studies published in the peer-reviewed literature (OR = 1.56; 95% CI, 1.19–2.04; *I*^2^ = 0%).

Exposure to residential insecticides during childhood was also positively associated with childhood leukemia (OR = 1.61; 95% CI, 1.33–1.95; *I*^2^ = 0%) when combining results from seven original studies (Figure 3B, Table 3). With restriction to studies of higher total methodological quality, the magnitude of the association was reduced and was no longer significant (OR = 1.36; 95% CI, 0.84–2.21; *I*^2^ = 30%). Our findings were similar when evaluating population-based studies (OR = 1.48; 95% CI, 1.03–2.11; *I*^2^ = 40%), studies of ALL (OR = 1.35; 95% CI, 0.76–2.38; *I*^2^ = 51%), and studies with results for outdoor insecticide use only (OR = 1.43; 95% CI, 0.71–2.86; *I*^2^ = 78%). Sensitivity analysis using an alternate exposure metric for Leiss and Savitz (1995) (using home extermination for insects as opposed to pest strips for insects in the home) decreased the magnitude of the association and also increased heterogeneity (OR = 1.29; 95% CI, 0.84–1.97; *I*^2^ = 62%). ORs were elevated, however, with recent year of publication (OR = 1.70; 95% CI, 1.28–2.27; *I*^2^ = 0%) and among studies published in the peer-reviewed literature (OR = 1.73; 95% CI, 1.41–2.12; *I*^2^ = 0%).

Finally, we observed no association between exposure to residential herbicides during childhood and childhood leukemia when combining results for four studies overall (OR = 0.96; 95% CI, 0.59–1.58; *I*^2^ = 72%) (Figure 3C, Table 4). We also observed substantial heterogeneity. Sensitivity analysis using alternate exposure indices for Ma et al. (2002) (using year 2 of childhood as opposed to year 1) and for Davis (1991) (using garden herbicide use between 7 months of age and age at diagnosis as opposed to yard herbicides from 0 to 6 months of age) resulted in a summary OR for childhood leukemia of 1.38 (95% CI, 1.10–1.72; *I*^2^ = 0%).

## Discussion

In this meta-analysis, we examined previous observational epidemiologic studies of the association between residential pesticide exposure during critical exposure time windows (preconception, pregnancy, childhood) and childhood leukemia. Overall, exposure to residential pesticides during pregnancy was positively associated with childhood leukemia. We observed the strongest association for insecticides, with little evidence of heterogeneity. Results for childhood exposures were less clear. Although we observed overall positive and significant associations for exposure to unspecified pesticides and insecticides during childhood, for insecticides the association attenuated among studies with higher total methodological quality. Few studies examined childhood exposure to herbicides, and we observed no overall positive association. Although we also examined preconceptional residential pesticide exposure, only two studies had clearly defined exposure time windows on which to base an assessment of the effect of this type of exposure on childhood leukemia. We obtained some differences in results in subgroup analysis according to study quality, study design, and publication status or when using alternate exposure indices for some of the associations we observed.

Previous reviews have concluded that there is likely to be a positive association between pesticide exposure and childhood leukemia (Daniels et al. 1997; Infante-Rivard and Weichenthal 2007). Results from a companion article revealed positive associations between childhood leukemia and prenatal maternal occupational exposure to pesticides (Wigle et al. 2009). Occupational pesticide exposures are of greater magnitude compared with those from other sources (Bradman et al. 2005; Mandel et al. 2005; Sala et al. 1999). Summary ORs for the relation between prenatal maternal occupational exposure to pesticides and childhood leukemia were larger compared with those here, with an overall summary OR of 2.08 (95% CI, 1.51–2.88), reported for any pesticide exposure, and 2.72 (95% CI, 1.47–5.04), reported for insecticide exposure, lending further credibility to the hypothesis.

Among the potential limitations of the present analysis is the possibility for publication bias. Although such bias can be difficult to assess, we found several small studies that were either unpublished (PhD dissertations) (Davis 1991; Dell 2004; Steinbuch 1994) or written in languages other than English (Fajardo-Gutierrez et al. 1993; Kishi et al. 1993). The magnitude of the association observed between unspecified pesticides and childhood leukemia tended to strengthen, and the heterogeneity reduce, on restriction to studies published in the peer-reviewed literature only.

Original studies may be subject to limitations related to exposure assessment and reporting. Typically, the quality of environmental epidemiology studies is influenced by the quality of exposure measurement (Hertz-Picciotto 1998). The studies in the present meta-analysis measured residential pesticide exposure entirely by parental report, and only in some instances were detailed data collected on specific types of pesticides or frequency of use. Although based on small numbers of exposed subjects, some limited evidence supports a positive exposure–response relationship between childhood leukemia and both pregnancy and childhood household pesticide or insecticide exposure (Alderton et al. 2006; Buckley et al. 1989; Infante-Rivard et al. 1999; Ma et al. 2002). Although there may be differential misclassification of exposure among cases, it has also been suggested that nondifferential misclassification may be of greater concern (Infante-Rivard and Jacques 2000). Although none of the studies we included here appear to have attempted to validate self-reported residential pesticide exposure information, Meinert et al. (1996, 2000) examined the risk of both childhood leukemia and solid tumors in the same study. They found a positive association between pesticides and childhood leukemia, but not solid tumors, possibly suggesting that the extent of recall bias by parents may be limited. The concordance of pesticide exposure among farmers, as measured via either self-report or biomonitoring, was poor (Arbuckle et al. 2004; Perry et al. 2006). Recently, Ward et al. (2009) examined exposure to persistent organochlorine pesticides in residential carpet dust samples in the Northern California Childhood Leukemia Study. They observed no positive associations with childhood leukemia for chlordane, DDT (dichlorodiphenyltrichloroethane) or its metabolite DDE (dichlorodiphenyldichloroethylene), methoxychlor, or pentachlorophenol concentrations.

Studies differed in the precise exposure time windows captured and reported. Some studies reported results only for all time windows combined, which may obscure the potential association linked to specific exposure time windows; however, because high correlations have been found between pesticide exposures in different exposure time windows (Alderton et al. 2006; Buckley et al. 1989), the extent to which such obfuscation might occur is difficult to determine. Sensitivity analyses that included studies reporting results in wide or ill-defined exposure time windows tended to increase the degree of heterogeneity we observed, as quantified by the *I*^2^ statistic. In models comparing pesticide exposures occurring during pregnancy, in childhood, and in both pregnancy and childhood, Menegaux et al. (2006) observed the strongest associations with childhood leukemia when exposures were experienced during both exposure time windows, as opposed to during one exposure time window only.

Exposure to different types of pesticides may also be correlated, and few studies have attempted to disentangle the independent effects of specific pesticides. Menegaux et al. (2006) incorporated different insecticide exposures simultaneously and found that the positive associations remained. Lowengart et al. (1987) reported that the positive associations observed for parental exposure to either household pesticides or garden pesticides/herbicides during pregnancy remained after mutual adjustment. Davis (1991) reported little change in pesticide ORs after adjustment for other pesticide use. However, Rudant et al. (2007) reported that associations with paternal pesticide use were confounded by maternal use. Residential pesticides also represent only one potential pathway through which parental or childhood pesticide exposure may occur, with food, occupation, and the transport of agricultural pesticides representing other potentially important exposure pathways (Lu et al. 2008; National Research Council 1993; Reynolds et al. 2005; Ritz and Rull 2008; Ward et al. 2009). Among studies of residential pesticides that also collected data on maternal occupational pesticide exposures, the prevalence of occupational pesticide exposure was low, and there is little information on the potential interrelationships of occupational and residential pesticide exposures for childhood leukemia. However, Rudant et al. (2007) reported that excluding children with occupationally exposed parents did not change results, and Buckley et al. (1989) reported that the positive associations observed for residential pesticide exposure remained in multivariate models containing parental occupational pesticide exposure. Residential pesticides may be an important exposure source even in agricultural areas (Quandt et al. 2004).

In terms of other potential confounding variables, as noted above, most studies examined or adjusted for at least a range of sociodemographic and maternal characteristics. Leiss and Savitz (1995) also considered magnetic field exposure. Menegaux et al. (2006) examined early common infections, child care attendance, and residence near a gas station/garage as potential confounders, with no change in results. Rudant et al. (2007) also reported that early infections and daycare attendance did not change results for residential pesticides. Lowengart et al. (1987) reported that the positive associations observed for both residential pesticide exposure and paternal occupational exposure to chlorinated solvents remained after mutual adjustment. Among the studies that assessed the potential influence of maternal or childhood X-ray exposures, Lowengart et al. (1987) and Fajardo-Gutierrez et al. (1993) reported no change in findings; however, Dell (2004) reported that the positive association observed between pregnancy exposure to yard pesticides and childhood leukemia disappeared after adjusting for maternal X-ray exposure and use of antibiotics during pregnancy.

For childhood leukemia, the pregnancy exposure time window may be of particular importance (Belson et al. 2007). Most childhood leukemia cases occur in the first few years of life (Agha et al. 2006). Most childhood leukemia cases have gross chromosomal abnormalities, including translocations; however, little is known regarding their underlying cause (Wiemels 2008). A study of routinely collected blood samples in neonates revealed leukemia clones with specific chromosomal translocations in children who later developed ALL (Gale et al. 1997). Preleukemic clones may persist throughout childhood and may require postnatal exposures for leukemia progression (Maia et al. 2004). In a small study of infants born in an agricultural region in the Philippines, the prevalence of a common AML translocation [t(8;21)] in cord blood samples was about 2-fold higher among those with detectable meconium levels of the methylcarbamate insecticide propoxur (Lafiura et al. 2007; Raimondi et al. 1999). The prenatal origin of AML may be less frequent than that of ALL (Burjanivova et al. 2006).

## Conclusions

This systematic review and meta-analysis reveals positive associations between exposure to residential pesticides in pregnancy and childhood and childhood leukemia, with the strongest associations observed for insecticides. Further work is needed to confirm previous findings based on self-report, to better describe potential exposure–response relationships, to assess specific pesticides and toxicologically related subgroups of pesticides in more detail, and to assess the potential role of preconceptional paternal exposures. Large prospective studies of children with biomonitoring data and discovery of biomarkers of past exposure (especially for rapidly excreted pesticides) would aid in this regard (Metayer and Buffler 2009). Additional studies are needed in order to better understand potential mechanisms of action and gene–pesticide interactions. In terms of precautionary public health implications, cosmetic pesticide bylaws implemented in various Canadian jurisdictions typically do not address the use of pesticides indoors or for essential purposes, such as to intervene in a health hazard or infestation to property. Further consideration of the need to reduce prenatal and childhood exposure to residential pesticides may be warranted.

## Figures and Tables

**Table 1:** Characteristics of included studies.

Reference, country	Cases/controls	Case definition	Age	Case recruitment	Control selection	Matching	Participation rate (cases/controls)
Hospital-based case–control studies							
Schwartzbaum et al. 1991, USA	629/72	ALL and ANLL	4.6 and 7.4 median years	Admitted to St. Jude’s Children’s Research Hospital 1979–1986	Rhabdomyosarcoma cases in same hospital	—	65.8^a^
Fajardo-Gutierrez et al. 1993, Mexico	81/154	Leukemia	8.3–8.5 mean years	Two hospitals in Mexico City	Noncancer hospital and community controls	Age, region	94/97
Kishi et al. 1993,^b^ Japan	77/158	ALL	< 15 years	Children’s Cancer Registry from four hospitals in Hokkaido 1980–1990	Noncancer inpatients in same or related hospital	Age, sex	—
Dell 2004, USA	49/97	Leukemia	< 18 years	Pennsylvania Cancer Registry from Children’s Hospital of Pittsburgh 1995–2000	Emergency department (2003), and parents from Health and Risk trial, and convenience sample	Age, sex, race	71/83
Alderton et al. 2006, USA/Canada	158/173	ALL and AML with Down syndrome	< 20 years	Children’s Oncology Group 1997–2002	Physicians of Down syndrome cases	Age	75/81
Menegaux et al. 2006, France	280/288	ALL and ANLL	< 15 years	Hospitalized in Lille, Lyon, Nancy, or Paris 1995–1999	Orthopedic and emergency department in same hospital	Age, sex, ethnic origin	99/99
Pombo-de-Oliveira et al. 2006, Brazil	202/440	IAL	< 22 months	15 institutions in 10 Brazil States 1999–2005	Hospitalized controls with severe life- threatening conditions	Age	96/95

Population-based case–control studies							
Lowengart et al. 1987, USA	123/123	ALL and ANLL	< 11 years	Los Angeles County Cancer Surveillance Program 1980–1984	Friends of cases and random digit dialing	Age, sex, race, Hispanic ethnicity	79/–
Buckley et al. 1989, USA/Canada	204/204	ANLL	< 18 years	Children’s Cancer Study Group 1980–1984	Random digit dialing	Age, race, region	83/85
Davis 1991, USA	71/85	ALL	< 11 years	Missouri Cancer Registry 1985–1989	Friends of cases	Age, sex	96/97
Kishi et al. 1993,^b^ Japan	103/264	ALL	< 15 years	Children’s Cancer Registry from four hospitals in Hokkaido 1980–1990	Same Health Region	Age, sex	—
Steinbuch 1994, USA	271/322	AML	< 18 years	Children’s Cancer Study Group 1989–1993	Random digit dialing	Age, race, region	93/81
Leiss and Savitz 1995, USA	—/222	Leukemia	< 15 years	Colorado Central Cancer Registry 1976–1983	Random digit dialing	Age, sex, region	71^c^/80
Meinert et al. 1996, Germany	173/220	ALL and ANLL	< 15 years	German Childhood Cancer Registry 1988–1992	Population-weighted sampling scheme, local and state controls	Age, sex, region	78/71^d^
Infante-Rivard et al. 1999, Canada	491/491	ALL	< 10 years	Tertiary care centers in Quebec 1980–1993	Family allowance files	Age, sex, region	96/84
Meinert et al. 2000, Germany	1,184/2,588	ALL and ANLL	< 15 years	German Childhood Cancer Registry 1992–1994, and from 1980–1994 for nuclear installation part	Lists of local resident registration offices	Age, sex, region	77^c^/63
Ma et al. 2002; Ma 2001, USA	162/162	ALL and ANLL	< 15 years	Major clinical centers in Northern California 1995–1999	Statewide birth certificate files	Age, sex, region, mother’s race, Hispanic ethnicity	83/69
Rudant et al. 2007, France	764/1,681	ALL and AML	< 15 years	Pediatric oncology centers and National Registry of Childhood Blood Malignancies 2003–2004	National telephone directory	Age, sex	91/71

Abbreviations: ANLL, acute nonlymphoblastic leukemia; IAL, infantile acute leukemia.

a Overall participation rate.

b The study of Kishi et al. (1993) is presented in both table sections because results are presented in the review for both hospital- and population-based controls.

c Participation rate for all cancer cases.

d Local controls.

**Table 2:** Random effects summary ORs (95% CIs) for the relation between childhood leukemia and exposure to unspecified residential pesticides by exposure time window.

	Pregnancy			Childhood		
Subgroup	Summary OR (95% CI)	*I*^2^ (%)	No. of included studies	Summary OR (95% CI)	*I*^2^ (%)	No. of included studies
Unspecified pesticides^a^	1.54 (1.13–2.11)	66	11	1.38 (1.12–1.70)	4	9
High total quality score	1.56 (1.08–2.27)	73	7	1.40 (1.05–1.85)	18	6
High external validity score	1.44 (0.94–2.20)	79	6	1.29 (0.87–1.93)	39	5
High exposure measurement score	1.72 (1.22–2.41)	0	5	1.44 (0.94–2.18)	34	5
High confounding score	1.66 (1.05–2.63)	79	6	1.48 (1.14–1.93)	0	4
Hospital based^b^	2.13 (0.89–5.06)	39	3	1.54 (1.07–2.22)	0	3
Population based	1.54 (1.10–2.16)	71	9	1.34 (1.03–1.75)	17	7
ALL^c^	2.04 (1.54–2.68)	19	5	1.40 (0.90–2.16)	32	4
AML	1.44 (0.81–2.59)	80	3	1.71 (0.77–3.80)	41	2
Indoor use	1.86 (1.25–2.77)	9	4	1.56 (1.02–2.39)	7	3
Outdoor use	1.50 (0.98–2.32)	31	5	1.40 (1.05–1.87)	0	4
Maternal use^d^	2.07 (1.62–2.64)	19	5	—	—	—
Year published (≥ 2000)	2.17 (1.85–2.53)	0	4	1.55 (1.14–2.12)	0	3
Peer-reviewed publication	1.81 (1.37–2.39)	36	8	1.56 (1.19–2.04)	0	6
Removing extreme ORs^e^	1.54 (1.11–2.13)	69	9	1.42 (1.15–1.75)	0	7
Removing highest weight^f^	1.40 (1.05–1.86)	34	9	1.38 (1.06–1.80)	16	8
Including wide/ill-defined exposure time windows^g^	1.51 (1.12–2.03)	79	13	1.35 (1.11–1.63)	48	12

a Where studies used multiple indices of exposure categories, the highest was selected, except for Dell (2004), which did not collect frequency information for all control groups. Where results were reported for leukemia overall as well as for specific cell types, the overall results were selected here. Where results were reported for indoor or outdoor pesticide use only, the indoor value was used here. Where results were reported for either owner-applied or professionally applied pesticides, the owner-applied value was used here. For Kishi et al. (1993), we selected results using population controls, except for the subgroup of hospital-based studies. For Buckley et al. (1989), unmatched OR of 1.47 (95% CI, 0.72–3.04) was used, calculated by collapsing the two highest exposure categories for pregnancy exposure. For the childhood time window, where studies reported results for different childhood time periods, the earliest was selected [for Ma et al. (2002), results for year 1 were used; for Davis (1991), results for 0–6 months were used; for Leiss and Savitz (1995), results from birth to 2 years before diagnosis were used].

b OR for Kishi et al. (1993) corrected to 1.80 for hospital controls, childhood exposure.

c Using results for ALL, instead of overall leukemia, for Ma et al. (2002).

d Unmatched OR of 3.52 (95% CI, 1.11–11.11) calculated from data in Lowengart et al. (1987).

e Removing studies with the highest and lowest ORs.

f Removing the study (or studies, in the case where there are two with identical values) with the highest weight in analysis.

g Including studies with wide or ill-defined exposure time windows.

**Table 3:** Random effects summary ORs (95% CIs) for the relation between childhood leukemia and exposure to residential insecticides by exposure time window.

	Pregnancy			Childhood		
Subgroup	Summary OR (95% CI)	*I*^2^ (%)	No. of included studies	Summary OR (95% CI)	*I*^2^ (%)	No. of included studies
Insecticides^a^	2.05 (1.80–2.32)	0	8	1.61 (1.33–1.95)	0	7
High total quality score	2.00 (1.72–2.33)	0	5	1.36 (0.84–2.21)	30	4
High external validity score	1.98 (1.56–2.50)	18	5	1.38 (0.80–2.38)	58	3
High exposure measurement score	1.79 (1.22–2.62)	0	3	1.36 (0.84–2.21)	30	4
High confounding score	2.05 (1.75–2.40)	0	4	1.36 (0.84–2.21)	30	4
Hospital based	2.05 (1.60–2.63)	0	2	1.74 (1.29–2.35)	0	3
Population based	2.04 (1.76–2.37)	0	6	1.48 (1.03–2.11)	40	4
ALL^b^	2.14 (1.83–2.50)	0	4	1.35 (0.76–2.38)	51	3
AML^c^	1.85 (1.29–2.64)	0	2	—	—	—
Indoor use only	1.90 (0.61–2.23)	0	4	1.59 (1.27–1.99)	8	6
Outdoor use only	1.54 (0.86–2.74)	36	4	1.43 (0.71–2.86)	78	3
Maternal use	2.02 (1.74–2.35)	0	4	—	—	—
Year published (2000 and later)	2.09 (1.82–2.39)	0	4	1.70 (1.28–2.27)	0	3
Peer-reviewed publication	2.12 (1.86–2.42)	0	6	1.73 (1.41–2.12)	0	5
Removing extreme ORs^d^	2.05 (1.80–2.34)	0	6	1.72 (1.40–2.11)	0	5
Removing highest weight^e^	1.98 (1.64–2.39)	0	7	1.47 (1.01–2.13)	19	5
Including unspecified, indoor pesticides^f^	2.02 (1.78–2.29)	0	11	1.63 (1.35–1.98)	0	11
Including wide/ill-defined exposure time windows^g^	1.81 (1.48–2.21)	46	10	1.44 (1.25–1.67)	15	8

a Where studies used multiple indices of exposure categories, the highest was selected, except for Dell (2004), which did not collect frequency information for all control groups. Where results were reported for leukemia overall as well as for specific cell types, the overall results were selected here. Where results were reported for indoor or outdoor insecticide use only, the indoor value was used. Where results were reported for either owner-applied or professionally applied insecticides, the owner-applied value was used here. For Pombo-de-Oliveira et al. (2006), personal correspondence with the study author (13 March 2008) corrected the upper CI reported in the published article from 2.13 to 2.95 and confirmed that pesticide exposure was mainly insecticide exposure. For the childhood time window, where studies reported results for different childhood time periods, the earliest was selected [for Ma et al. (2002), results for year 1 used; for Davis (1991), results for 0–6 months used here; for Leiss and Savitz (1995), results from birth to 2 years before diagnosis used here]. For Fajardo-Gutierrez et al. (1993), personal correspondence with the study author (28 May 2008) confirmed exposure was postnatal exposure to insecticides in the home.

b Using results for ALL, instead of overall leukemia, for Ma et al. (2002) and Rudant et al. (2007).

c Using results for AML, instead of overall leukemia, for Rudant et al. (2007).

d Removing studies with the highest and lowest OR.

e Removing the study (or studies, in the case where there are two with identical values) with the highest weight in analysis.

f Including studies that reported indoor unspecified pesticide use.

g Including studies with wide or ill-defined exposure time windows.

**Table 4:** Random effects summary ORs (95% CIs) for the relation between childhood leukemia and exposure to residential herbicides by exposure time window.

	Pregnancy			Childhood		
Subgroup	Summary OR (95% CI)	*I*^2^ (%)	No. of included studies	Summary OR (95% CI)	*I*^2^ (%)	No. of included studies
Herbicides^a^	1.61 (1.20–2.16)	0	5	0.96 (0.59–1.58)	72	4
High total quality score	1.57 (1.17–2.11)	0	4	0.81 (0.40–1.64)	80	3
High external validity score	1.56 (1.11–2.18)	0	3	0.81 (0.24–2.77)	85	2
High exposure measurement score	1.68 (1.05–2.68)	0	3	0.81 (0.40–1.64)	80	3
High confounding score	1.58 (1.15–2.18)	0	3	1.04 (0.52–2.05)	80	2
Population based	1.57 (1.17–2.11)	0	4	0.81 (0.40–1.64)	80	3
ALL^b^	1.73 (1.28–2.35)	0	4	0.85 (0.43–1.66)	78	3
Maternal use	1.54 (1.12–2.12)	0	4	—	—	—
Year published (2000 and later)	1.57 (1.14–2.17)	0	3	0.99 (0.50–1.96)	68	2
Peer-reviewed publication	1.62 (1.18–2.23)	0	4	1.16 (0.77–1.75)	61	3
Removing extreme ORs^c^	1.68 (1.05–2.68)	0	3	0.99 (0.50–1.96)	68	2
Removing highest weight^d^	1.77 (1.12–2.80)	0	4	0.79 (0.40–1.53)	68	3
Including unspecified, outdoor pesticides^e^	1.56 (1.23–1.99)	0	8	1.06 (0.73–1.52)	60	6
Including wide/ill-defined exposure time windows^f^	—	—	—	1.14 (0.94–1.39)	56	9

a Where studies used multiple indices of exposure categories, the highest was selected, except for Dell (2004), which did not collect frequency information for all control groups. Where results were reported for leukemia overall as well as for specific cell types, the overall results were selected here. For the childhood time window, where studies reported results for different childhood time periods, the earliest was selected [for Ma et al. (2002), results for year 1 used here; for Davis (1991), results for 0–6 months].

b Using results for ALL, instead of overall leukemia, for Ma et al. (2002) and Rudant et al. (2007).

c Removing studies with the highest and lowest OR.

d Removing the study (or studies, in the case where there are two with identical values) with the highest weight in analysis.

e Including studies that reported outdoor unspecified pesticide use. For Leiss and Savitz (1995), the earliest time window from birth to 2 years before diagnosis used for childhood analysis.

f Including studies with wide of ill-defined exposure time windows.

## Appendix 1 Definitions of exposure time windows reported in individual studies

### Preconception

#### Well defined

- 3 months before conception (Ma et al. 2002)
- 2 years before conception (Dell 2004)

#### Wide/ill defined

- 3 months before pregnancy to lactation (Pombo-de-Oliveira et al. 2006)
- 2 years before birth to date of diagnosis/reference date (Meinert et al. 1996)
- 1 year before pregnancy to reference date (Schwartzbaum et al. 1991)

### Pregnancy

#### Well defined

- 3 months before birth (Leiss and Savitz 1995)
- Conception to birth (Buckley et al. 1989; Davis 1991; Dell 2004; Kishi et al. 1993; Lowengart et al. 1987; Ma et al. 2002; Menegaux et al. 2006; Rudant et al. 2007)
- 1 month before pregnancy to birth (Infante-Rivard et al. 1999; Steinbuch 1994)
- Conception to lactation (maternal) (Lowengart et al. 1987)
- 1 month before pregnancy, pregnancy, and lactation (Alderton et al. 2006)
- 3 months before pregnancy to lactation (Pombo-de-Oliveira et al. 2006)

#### Wide/ill defined

- 2 years before birth to date of diagnosis/reference date (Meinert et al. 1996)
- Year of birth to diagnosis/reference date (Meinert et al. 2000)

### Childhood

#### Well defined

- End of lactation to date of diagnosis/reference date (Alderton et al. 2006)
- Birth to date of diagnosis/reference date (Buckley et al. 1989; Dell 2004; Fajardo-Gutierrez et al. 1993^a^; Infante-Rivard et al. 1999; Menegaux et al. 2006; Schwartzbaum et al. 1991; Steinbuch 1994)
- Birth to 2 years before diagnosis, and 2 years before diagnosis to diagnosis (Leiss and Savitz 1995)
- Years 1, 2, and 3 after birth (Ma et al. 2002)
- Onset of disease (Kishi et al. 1993)
- Birth to 6 months, and 7 months to date of diagnosis/reference date (Davis 1991)

#### Wide/ill defined

- Pregnancy and childhood, paternal (Rudant et al. 2007)
- 2 years before birth to date of diagnosis/reference date (Meinert et al. 1996)
- Year of birth to diagnosis/reference date (Meinert et al. 2000)
- 1 year before pregnancy to reference date (Steinbuch 1994)
